# No-Signaling in Steepest Entropy Ascent: A Nonlinear, Non-Local, Non-Equilibrium Quantum Dynamics of Composite Systems Strongly Compatible with the Second Law

**DOI:** 10.3390/e27101018

**Published:** 2025-09-28

**Authors:** Rohit Kishan Ray, Gian Paolo Beretta

**Affiliations:** 1 Department of Material Science and Engineering, Virginia Tech, Blacksburg, VA 24061, USA; 2 Center for Theoretical Physics of Complex Systems, Institute for Basic Science (IBS), Daejeon 34126, Republic of Korea; 3 Department of Mechanical and Industrial Engineering, University of Brescia, 25123 Brescia, Italy; gianpaolo.beretta@unibs.it

**Keywords:** non-linear quantum thermodynamics, entropy production, no-signaling, steepest entropy ascent, local perception operators

## Abstract

Lindbladian formalism models open quantum systems using a ‘bottom-up’ approach, deriving linear dynamics from system–environment interactions. We present a ‘top-down’ approach starting with phenomenological constraints, focusing on a system’s structure, subsystems’ interactions, and environmental effects and often using a non-equilibrium variational principle designed to enforce strict thermodynamic consistency. However, incorporating the second law’s requirement—that Gibbs states are the sole stable equilibria—necessitates nonlinear dynamics, challenging no-signaling principles in composite systems. We reintroduce ‘local perception operators’ and show that they allow to model signaling-free non-local effects. Using the steepest-entropy-ascent variational principle as an example, we demonstrate the validity of the ‘top-down’ approach for integrating quantum mechanics and thermodynamics in phenomenological models, with potential applications in quantum computing and resource theories.

## 1. Introduction

The study of open quantum systems broadly falls into two distinct approaches. The dominant method involves the Gorini–Kossakowski–Lindblad–Sudarshan (GKLS) master equation, typically under the assumption of weak coupling between a quantum system and its environment (for recent reviews, see [1,2]). This approach derives thermodynamically consistent reduced system dynamics starting from the Schrödinger equation combined system-environment evolution and subsequently tracing out environmental degrees of freedom [3,4,5]. We refer to this widely adopted strategy as the ‘bottom-up’ approach. Its effectiveness relies on idealized assumptions regarding the environment and the careful construction of appropriate Kraus operators, and it typically presumes that the initial state of the system-environment composite is factorizable.

In contrast, an alternative yet less explored methodology exists: the phenomenological or ‘top-down’ approach. This paradigm directly posits a general local dynamical law for the system’s density operator based primarily on thermodynamic and phenomenological considerations. It inherently accommodates strongly coupled subsystems and composite structures. Crucially, it explicitly incorporates the thermodynamic requirement that Gibbs states are the only conditionally stable equilibrium states [6,7], necessitating a departure from strictly linear evolution. Simmons and Park [6] demonstrated early on that nonlinearity is essential to achieve a consistent integration of quantum mechanics and thermodynamics. It is important to clarify here that our approach is distinct from the nonlinear quantum mechanics (NQM) recently subjected to stringent experimental tests [8,9]; rather, it represents a phenomenological route toward thermodynamically consistent, irreversible and non-equilibrium quantum dynamical models.

Recent experimental advances underscore the practical necessity for top-down phenomenological models, especially in scenarios where detailed environmental modeling is inaccessible or impractical [10]. Such models naturally describe ‘apparent decoherence’ stemming from unknown environmental interactions, employing minimal empirical parameters. Moreover, they conveniently handle situations where multiple conserved quantities, possibly non-commuting, are relevant at the local level [11].

Among the existing top-down formalisms, the Steepest Entropy Ascent (SEA) framework has emerged as particularly powerful and conceptually robust. Originating from the foundational works of Hatsopoulos and Gyftopoulos [12,13,14,15], and subsequently developed extensively by Beretta [16,17,18,19,20,21,22,23,24], SEA integrates thermodynamic irreversibility into quantum dynamics from first principles. This formalism has been successfully employed across diverse quantum systems [25,26,27], including quantum computational scenarios [28,29]. Nevertheless, despite its robustness and conceptual appeal, a rigorous demonstration of SEA’s compatibility with fundamental quantum principles, particularly the no-signaling condition, has thus far remained underexplored [22,23,24,26].

This paper explicitly fills this critical gap. We rigorously prove that SEA dynamics inherently satisfy the no-signaling condition, even though its equations of motion incorporate nonlinearities and nonlocal structures. To this end, we introduce and utilize Local Perception Operators (LPOs), a conceptual tool that ensures subsystem locality in a nonlinear dynamical context. We establish the invariance of LPOs under local unitary transformations, which guarantees that no signaling between non-interacting subsystems emerges from the SEA dynamics. We further substantiate our claims through nontrivial illustrative examples. While the primary objective is to demonstrate that SEA-type dynamics respect no-signaling, we also take this opportunity to clarify the foundational motivation for invoking the SEA framework in the first place. In doing so, we show how it resolves subtle but important paradoxes that often go unaddressed in conventional bottom-up approaches.

The paper is structured as follows. Section 2 formally defines signaling and explores its relationship with nonlinearity. In Section 3, we revisit the philosophical motivations that led to the inception of SEA dynamics. Section 4 discusses conceptual issues surrounding quantum state individuality that nonlinear dynamics help resolve. Section 5 introduces a measure-theoretic representation for mixed ensembles, facilitating the rigorous handling of nonlinear dynamics. The Local Perception Operators (LPOs) are defined and analyzed in Section 6, and their critical role in establishing no-signaling is rigorously demonstrated in Section 7. The composite-system SEA equation is developed structurally in Section 8, followed by a variational derivation in Section 9 and Section 10. Section 11 presents explicit numerical examples, and Section 12 summarizes our findings.

## 2. No-Signaling and Nonlinearity

Quantum mechanics (QM), as described by the Schrödinger-von Neumann formalism, is traditionally characterized by its linearity in state space and time evolution. Mean values of global and local properties and their time evolutions under unitary (and completely positive) dynamics are linear functionals of the density operator. This linearity implies no-cloning, first shown by Park [30] and later rediscovered by Ghirardi [31], Wootters and Zurek [32], and Dieks [33]. Linearity also implies no-signaling [34,35,36,37]. However, Weinberg questioned this linear characteristic of QM [38]. He introduced nonlinearity into the operator formalism of the Schrödinger equation while preserving order-one homogeneity. This was one of the earliest examples of what is now broadly referred to as nonlinear quantum mechanics (NQM). Gisin [39] showed that such formalism could facilitate faster-than-light communication, viz., signaling. Polchinski [40] proposed an alternative method to prevent signaling by restricting local evolutions in order to depend solely on the corresponding reduced density operators. However, this formalism still allowed signaling between different branches of the wave function (Everett telephone) [40]. Wódkiewicz and Scully [41] analyzed the nonlinear evolution of two-level atoms, with solutions later shown to depend on interpretation [42]. Czachor also showed that a nonlinear operator induces mobility phenomena (non-conservation of the inner-product of two pure states) [43].

Nonlinearity introduced via stochastic QM through Lindblad operator formalism [44] for open quantum systems has been shown to respect no-signaling. Weinberg-inspired nonlinearities, interestingly, enable faster algorithms to solve *NP*-complete problems in polynomial time [45]. Later work [46] demonstrated that nonlinearity in QM can be incorporated without violating no-signaling, as long as time evolution is nonlinear while state space and operators remain linear. More recently, convex quasilinear maps [47,48] were shown to support nonlinear QM dynamics without signaling, preserving key features of QM. Rembieliński and Caban [47] identified this as the minimal allowable deviation from QM’s linear structure. In a recent work, Kaplan and Rajendran [49] showed that, in a low-temperature limit, the non-linearity introduced in quantum field theory results in causal nonlinear quantum mechanics, which is also another version of NQM.

**Figure 1:**
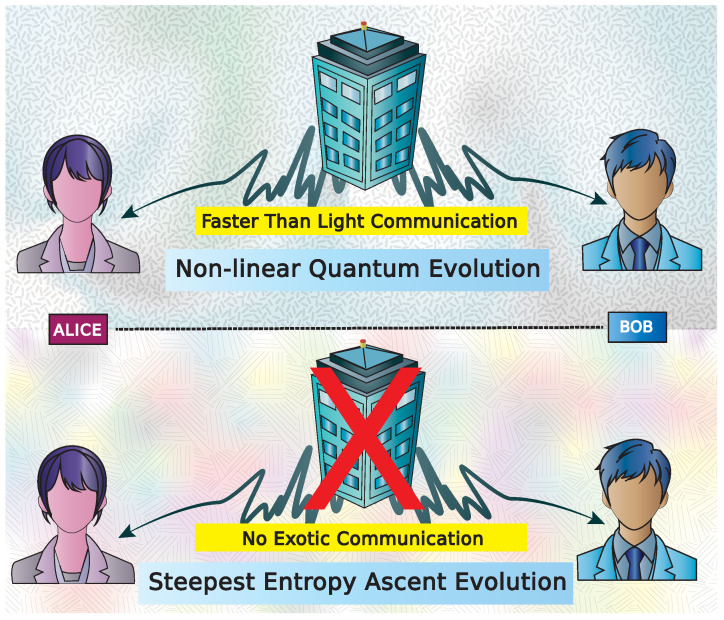
Schematic representation of no-signaling situation in the case of nonlinear quantum evolution. Alice and Bob are correlated but are non-interacting in a nonlinear quantum theory (we imply the nonlinearities via the texture of the embedding background in the schematic). In the top part, a non-linear quantum evolution as described by Polchinski [40] or Gisin [39] may entail faster-than-light communication in some form (denoted as the telephone booth; see text for details). In the bottom part, instead, the embedding nonlinear steepest-entropy-ascent evolution cannot establish a similar exotic communication.

Therefore, introducing nonlinearity in quantum mechanics (QM) can lead to exotic interactions that are difficult to justify physically. Nonlinear theories, as inspired by the Weinberg approach, allow for entropy oscillation [43]. The stochastic, jump-induced mixing of pure states [44] offers a basis for developing a thermodynamically consistent nonlinear master equation for the density operator of an open system. However, the search for theoretically consistent nonlinear models of quantum thermodynamics remains an open challenge. This is where we claim—and later demonstrate in the following sections—that the ‘top-down’ approach of SEA resolves the signaling problem entirely (pictorially sketched in Figure 1). From a philosophical perspective, SEA also addresses other conceptual conundrums, in particular the Schrödinger-Park paradox [50,51], as was already discussed by Park [52] and Beretta [53].

The no-signaling condition, as noted in [46], is typically enforced by requiring that, in the absence of mutual interactions between subsystems A and B, the evolution of A’s local observables depends solely on its reduced state. Formally, we express this as follows:

(1) $$\frac{d\rho_{J}}{dt}\;=\;f\left(\rho_{J}\right),$$

where $\rho_{J}$ is the reduced density operator (local state) of subsystem *J*. The SEA formalism, however, adopts a less restrictive perspective [22]. It requires that, if A and B are non-interacting, the law of evolution must not permit a local operation within B to influence the time evolution of A’s local (reduced, marginal) state. Consequently, the condition $\rho_{A}=\rho_{A}^{\prime }$, applied to the two different states $\rho \neq \rho_{A}⊗\rho_{B}$ and $\rho^{\prime }=\rho_{A}⊗\rho_{B}$, does not necessitate $d\rho_{A}/dt=d\rho_{A}^{\prime }/dt$. This is because the local evolution can still be influenced by past interactions, such as existing entanglement and correlations, without violating the no-signaling principle. This highlights two key ideas: (1) analyzing local evolutions allows detection of correlations, but only those that can be classically communicated between subsystems, and (2) in the absence of interactions, nonlinear dynamics can cause correlations to diminish (spontaneous decoherence) but cannot create new correlations. We formally express this no-signaling condition as follows (see Section 8 for details),

(2) $$\frac{d\rho_{J}}{dt}\;=\;f\left(\rho_{J},{\left(C_{k}\right)}^{J}\right),$$

where ${\left(C_{k}\right)}^{J}$ are ‘local perception operators’ (LPO) (see Section 6 for their definition).

## 3. Philosophical Motivations Leading to SEA

Philosophically, SEA evolution was designed to conceive the irreversible equilibration and decoherence as a fundamental spontaneous dynamical feature, contrary to the coarse-graining approach. Entropy, the second law, and irreversibility could attain a more fundamental status as deterministic outcomes of quantal evolution [54], without contradicting standard QM. From the outset [16,17], nonlinearity was recognized as essential [6,55] for this purpose. While the prevalent notion was—and still is—that the second law is emerging and statistical, the pioneers of SEA believed that the many ‘knots of thermodynamics’ [55,56] could, at least conceptually, be untied by elevating it to a more fundamental stature. This motivated Hatsopoulos and Gyftopoulos (HG) to develop a unified theory of mechanics and thermodynamics [12,13,14,15]. They laid the foundation for what is now known as resource theory in quantum thermodynamics by extending ontological quantum states to include both pure and mixed density operators. The HG unified (resource) theory explicitly defines and fully characterizes the concept—later rediscovered as ‘ergotropy’ [57]—of maximum work extractable in a unitary process with cyclic parameter changes (theorems 3.2 and 3.3 in [13], which pioneered also the use of ‘majorization’ in quantum theory).

Beretta’s doctoral thesis [58] aimed to complete the HG theory with two key goals: (1) to design an equation of motion that establishes the second law of thermodynamics as a theorem, ensuring Gibbs states are the only conditionally stable equilibrium states; and (2) to develop an unambiguous statistical representation of heterogeneous ensembles formed by the statistical mixing of homogeneous (pure) ensembles as one-to-one with the full set of pure and mixed density operators.

The outcome of the first objective was the SEA formalism, including the introduction of local perception operators. SEA formalism proved to be both robust and versatile, making it a powerful tool for thermodynamically consistent non-equilibrium modeling. Its applications expanded beyond its original scope to include phenomenological models in areas such as decoherence [59], mesoscopic transport rheology [60,61], phase transitions [62], and quantum computing [27,28,29,63].

The second objective led to the representation of generic ensembles using measure-theoretic distributions over the set of ontological states. In the context of quantum thermodynamics, an “unambiguous statistical representation of heterogeneous ensembles” ensures a clear distinction between intrinsic quantum uncertainties and classical statistical mixing. This approach—detailed in Section 5 following [53,58]—represents heterogeneous ensembles as distributions over the full set of pure and mixed density operators, thus avoiding ambiguities associated with von Neumann’s statistical interpretation and resolving the Schrödinger-Park paradox.

## 4. Nonlinear Dynamics and the Ontic State Conundrum

From a foundational perspective, the HG approach of assigning ontological status to the full set of density operators, pure and mixed, offers a potential resolution to the Schrödinger–Park paradox concerning the ambiguity of individual quantum states [50,51,53]. As noted by Park [52], treating density operators as one-to-one with homogeneous ensembles resolves ambiguity under two conditions: (1) that they evolve unitarily for short times, with nonlinear dynamics dominating over longer periods; and (2) that a full tomography, even for heterogeneous preparations, must include both linear and nonlinear observables, such as entropy, adiabatic availability, ergotropy, and free energy.

The von Neumann prescription [64] (Chapter III) assigns a density operator to a statistical mixture of pure states, with mixing coefficients reflecting ignorance. However, Schrödinger [50] questioned this from the outset due to the non-unique decomposition of a mixed density operator into weighted sums of pure states. von Neumann’s approach irrecoverably blends quantal probabilities with non-quantal statistical weights, creating the ambiguity highlighted by Schrödinger and Park. To understand how a law of nonlinear evolution may resolve this issue, let ${\hat{\Pi }}_{1}^{0}$ and ${\hat{\Pi }}_{2}^{0}$ be two distinct homogeneous preparations. At time t=0, they generate two initial ensembles of identically prepared, strictly isolated, and non-interacting systems. The ‘hat’ on Π denotes the homogeneity of the preparation, and superscript ‘0’ refers to time t=0. According to the traditional statistical formulation by von Neumann, a preparation, $\hat{\Pi }$, is *homogeneous* if and only if it cannot be replicated using any statistical composition of two *different* preparations. The statistics of measurement outcomes from a homogeneous preparation are represented as a pure (idempotent) density operator, $\hat{\rho }$, satisfying $\hat{\rho }\;=\;{\hat{\rho }}^{2}$. Consider a third (heterogeneous) preparation, $\Pi_{3}^{0}$, obtained via the statistical mixture of ${\hat{\Pi }}_{1}^{0}$ and ${\hat{\Pi }}_{2}^{0}$ with a fixed weight, 0<w<1, so that, formally, we may write $\Pi_{3}^{0}=w\;{\hat{\Pi }}_{1}^{0}+\left(1-w\right)\;{\hat{\Pi }}_{2}^{0}$. To obtain quantum statistical mechanics (QSM) consistent with Gibbs–Boltzmann, Fermi–Dirac, and Bose–Einstein equilibrium distributions, von Neumann postulated that a heterogeneous preparation, such as $\Pi_{3}^{0}$, should be represented as a mixed density operator, $\rho_{3}^{0}\;=\;w\;{\hat{\rho }}_{1}^{0}+\left(1-w\right)\;{\hat{\rho }}_{2}^{0}$. This formulation, partly due to the linearity of quantum mechanics, has served as the foundation for successful frameworks such as equilibrium QSM and the Lindbladian theory of open systems.

These successes often lead us to overlook the Schrödinger–Park paradox or accept it as a seemingly enigmatic aspect of quantum theory. It arises when interpreting a heterogeneous ensemble. If we assume that each member of the ensemble is prepared in either state ${\hat{\rho }}_{1}^{0}$ (with probability *w*) or ${\hat{\rho }}_{2}^{0}$ (with probability 1−w), excluding all other states, we face a contradiction. The same mixed density operator, $\rho_{3}^{0}$, allows infinitely many alternative decompositions. For instance, the decomposition $\rho_{3}^{0}\;=\;w^{\prime }\;{\hat{\rho }}_{4}^{0}+\left(1-w^{\prime }\right)\;{\hat{\rho }}_{5}^{0}$ suggests that the ensemble consists only of ${\hat{\rho }}_{4}^{0}$ and ${\hat{\rho }}_{5}^{0}$, excluding ${\hat{\rho }}_{1}^{0}$ and ${\hat{\rho }}_{2}^{0}$. This inconsistency challenges the notion of individual states in a heterogeneous ensemble and complicates the interpretation of QSM. Under a linear evolution map for the homogeneous preparations, ${\hat{\Pi }}^{t}=L_{t}\left({\hat{\Pi }}^{0}\right)$, and mixing of homogeneous preparations with time-invariant weights, we have

(3) $$\Pi_{3}^{t}=w\;{\hat{\Pi }}_{1}^{t}+\left(1-w\right)\;{\hat{\Pi }}_{2}^{t}=w\;L_{t}\left({\hat{\Pi }}_{1}^{0}\right)+\left(1-w\right)\;L_{t}\left(\Pi_{2}^{0}\right)$$

and, via the linearity of $L_{t}$, $\Pi_{3}^{t}=L_{t}\left({\hat{\Pi }}_{3}^{0}\right)$. Thus, the von Neumann recipe gives $\rho_{3}^{t}\;=\;w\;{\hat{\rho }}_{1}^{t}+\left(1-w\right)\;{\hat{\rho }}_{2}^{t}$ and, for linear observables, it yields the correct statistics of measurements,

(4) $$\mathrm{Tr}\left(A\;\rho_{3}^{t}\right)\;=\;w\;\mathrm{Tr}\left(A\;{\hat{\rho }}_{1}^{t}\right)+\left(1-w\right)\;\mathrm{Tr}\left(A\;{\hat{\rho }}_{2}^{t}\right).$$

It also entails that, for an *N*-level system, a full tomography of a heterogeneous preparation requires the measurement of a quorum of only $N^{2}-1$ independent linear observables.

Instead, if the evolution map for homogeneous preparations is nonlinear, ${\hat{\Pi }}^{t}=N_{t}\left({\hat{\Pi }}^{0}\right)$, and the statistical mixing weights remain time-invariant, we obtain

(5) $$\Pi_{3}^{t}=w\;{\hat{\Pi }}_{1}^{t}+\left(1-w\right)\;{\hat{\Pi }}_{2}^{t}=w\;N_{t}\left({\hat{\Pi }}_{1}^{0}\right)+\left(1-w\right)\;N_{t}\left(\Pi_{2}^{0}\right)$$

and, in general, due to the nonlinearity of $N_{t}$, this does not satisfy $\Pi_{3}^{t}\neq N_{t}\left({\hat{\Pi }}_{3}^{0}\right)$. This contradicts the von Neumann prescription, which would assign the density operator $\rho_{3}^{t}\;=\;w\;\rho_{1}^{t}+\left(1-w\right)\;\rho_{2}^{t}$ to preparation $\Pi_{3}^{t}$ and $\rho_{3}^{0}\;=\;w\;\rho_{1}^{0}+\left(1-w\right)\;\rho_{2}^{0}$ to preparation $\Pi_{3}^{0}$. As noted by Rembieliński and Caban [47], a sufficient condition for a nonlinear map to be no-signaling is convex-quasilinearity. That is, it must always admit a $w^{\prime }$, with $0\leq w^{\prime }\leq 1$, such that $\rho_{3}^{t}\;=\;w^{\prime }\;\rho_{1}^{t}+\left(1-w^{\prime }\right)\;\rho_{2}^{t}.$ Ferrero et al. [46] made a similar observation but left its interpretation as an open problem.

The HG ontological hypothesis alleviates this interpretation issue. Regardless of how a density operator is decomposed, *w* and 1−w no longer represent epistemic ignorance. This is because both pure and mixed density operators have ontic status, meaning they represent homogeneous preparations.

## 5. Measure-Theoretic Representation of Statistics from Mixed Ensembles

To resolve the Schrödinger-Park paradox about individual states in QSM without contradicting the successes of QM, Beretta [58] proposed replacing the von Neumann prescription for measurement statistics from mixed ensembles with an unambiguous representation akin to classical statistical mechanics. The recipe assigns to every preparation Π (whether homogeneous or heterogeneous) a normalized *measure* $\mu_{\Pi }$ defined on the (ontic) quantal state domain P, which consists of the mathematical objects representing homogeneous preparations (homogeneous ensembles). The normalization condition is

(6) $$\mu_{\Pi }\left(P\right)\;=\;\int_{P}\mu_{\Pi }\left(d\rho \right)\;=\;1.$$

In usual von Neumann QSM, the quantal state domain $P_{\mathrm{QSM}}$ is the set of all one-dimensional projection operators (i.e., the idempotent density operators) defined on the Hilbert space of the system. Instead, the HG-unified theory assumes the broader quantal state domain $P_{\mathrm{HG}}$ consisting of the set of all possible density operators ρ, idempotent and not.

Among the measures defined on P, the *Dirac measures*, defined as follows, play a crucial role. Let E denote any subset of P, and then

(7) $$\delta_{\rho }\left(E\right)=\left\{\begin{array}{c} 1,\;\;\;\mathrm{if}\;\rho \in E\\ 0,\;\;\;\mathrm{if}\;\rho \notin E \end{array}\right.$$

The support of a Dirac measure, $\delta_{\tilde{\rho }}$, i.e., the subset of P for which it is nonzero, is a single point coinciding with the state operator $\tilde{\rho }$.

The statistical mixing of preparations, $\Pi_{3}=w\;\Pi_{1}+\left(1-w\right)\;\Pi_{2}$, is represented by the weighted sum of the corresponding measures,

(8) $$\mu_{\Pi_{3}}=w\;\mu_{\Pi_{1}}+\left(1-w\right)\;\mu_{\Pi_{2}}.$$

This representation removes ambiguities because of the following:

1. No Dirac measure can be decomposed into a weighted sum of different Dirac measures, satisfying von Neumann’s definition of homogeneous preparations.
2. Any measure has a unique decomposition into a weighted sum (or integral) of Dirac measures—removing the Schrödinger–Park paradox. This ensures that each member of a heterogeneous ensemble is in some well defined (albeit unknown) individual state.

The expected mean value of any physical observable represented by the (linear or nonlinear) functional *g* defined on P is given for preparation $\Pi_{3}=w\;\Pi_{1}+\left(1-w\right)\;\Pi_{2}$ by

(9) $$\begin{array}{cc} \; & {\bar{\langle g\rangle }}_{\Pi_{3}}=\int_{P}g\left(\rho \right)\;\mu_{\Pi_{3}}\left(d\rho \right)\\ & =\int_{P}g\left(\rho \right)\;\left[w\;\mu_{\Pi_{1}}\left(d\rho \right)+\left(1-w\right)\;\mu_{\Pi_{2}}\left(d\rho \right)\right]\\ & =w\;\int_{P}g\left(\rho \right)\;\mu_{\Pi_{1}}\left(d\rho \right)+\left(1-w\right)\;\int_{P}g\left(\rho \right)\;\mu_{\Pi_{2}}\left(d\rho \right). \end{array}$$

If the component preparations are homogeneous, i.e., if $\mu_{{\hat{\Pi }}_{1}}=\delta_{\rho_{1}}$ and $\mu_{{\hat{\Pi }}_{2}}=\delta_{\rho_{2}}$, then

(10) $$\begin{array}{cc} \; & {\bar{\langle g\rangle }}_{\Pi_{3}}\;=\;w\;\int_{P}g\left(\rho \right)\;\delta_{\rho_{1}}\left(d\rho \right)+\left(1-w\right)\;\int_{P}g\left(\rho \right)\;\delta_{\rho_{2}}\left(d\rho \right)\\ & =w\;g\left(\rho_{1}\right)+\left(1-w\right)\;g\left(\rho_{2}\right)\\ & =w\;{\langle g\rangle }_{{\hat{\Pi }}_{1}}+\left(1-w\right)\;{\langle g\rangle }_{{\hat{\Pi }}_{2}}\;. \end{array}$$

For example, for $P\;=\;P_{\mathrm{HG}}$ and $g\left(\rho \right)=-k_{B}\mathrm{Tr}\left(\rho \ln\rho \right)$, this gives the ‘proper’ expected value of measurements of the von Neumann entropy, i.e., the weighted sum of the entropies of the component homogeneous sub-ensembles.

It is noteworthy that, for observables represented by linear functionals on P, such as g(ρ)=Tr(Gρ), we have

(11) $$\begin{array}{cc} {\bar{\langle g\rangle }}_{\Pi_{3}} & =w\;\mathrm{Tr}\left(G\;\rho_{{\hat{\Pi }}_{1}}\right)+\left(1-w\right)\;\mathrm{Tr}\left(G\;\rho_{{\hat{\Pi }}_{2}}\right)\\ & =\mathrm{Tr}(G\;\left[w\;\rho_{{\hat{\Pi }}_{1}}+\left(1-w\right)\;\rho_{{\hat{\Pi }}_{2}}\right])\\ & \;=\;\mathrm{Tr}\left(G\;W_{\Pi_{3}}\right)\;=\;g\left(W_{\Pi_{3}}\right)\;, \end{array}$$

where $W_{\Pi_{3}}$ is the usual von Neumann statistical operator, defined by the weighted sum of the density operators representing the component homogeneous sub-ensembles,

(12) $$W_{\Pi_{3}}=w\;\rho_{{\hat{\Pi }}_{1}}+\left(1-w\right)\;\rho_{{\hat{\Pi }}_{2}}.$$

$W_{\Pi_{3}}$ is clearly an element of the convex completion, C(P), of the quantal state domain P. Notice that, in general $C\left(P_{\mathrm{QSM}}\right)=C\left(P_{\mathrm{HG}}\right)=P_{\mathrm{HG}}$, and for a qubit $P_{\mathrm{QSM}}$ and $P_{\mathrm{HG}}$ map to the Bloch sphere and the Bloch ball, respectively. An evaluation of the linear functional g(·)=Tr(G·) at $W_{\Pi_{3}}$ provides the correct expected mean value of measurements of the corresponding observable on the heterogeneous ensemble.

But $W_{\Pi_{3}}$ does not fully describe the heterogeneous preparation $\Pi_{3}$, because different preparations can yield the same linear tomography. In other words, the linear tomography—obtained by measuring the expected mean values $\bar{\langle q_{j}\rangle }$ of a quorum of $N^{2}-1$ independent linear observables $Q_{j}$ and solving $\bar{\langle q_{j}\rangle }=\mathrm{Tr}\left(Q_{j}\;W\right)$ for *W*—is insufficient to characterize a preparation. A decomposition of *W* into different weighted sums of density operators has no meaning in this theory, but it emphasizes that different heterogeneous preparations may result in the same linear tomography. Even within orthodox QSM ($P\;=\;P_{\mathrm{QSM}}$), resolving the intrinsic quantum probabilities of homogeneous preparations from the extrinsic uncertainties of mixing requires additional independent information beyond linear tomography.

The measure-theoretic description of preparations enables a statistical quantum theory in which both linear and nonlinear functionals of the density operator correspond to independently and directly measurable properties of a quantum system, such as entropy, ergotropy, and adiabatic availability. A nonlinear evolution equation for the density operators in P, i.e., for the homogeneous preparations, may provide additional nonlinear observables by measuring linear and nonlinear observables at different times. As already realized by Park [52], nonlinearity holds the promise of preserving and reintegrating the notion of an individual state into quantum theory.

## 6. Local Perception Operators (LPOs)

In linear QM, the system’s composition is specified by declaring the following:

1. The Hilbert space structure as the direct product H=⨂JJ¯=1MHJJ¯ of the subspaces of the *M* component subsystems.
2. The overall Hamiltonian operator H=∑JJ¯=1MHJJ¯⊗IJ¯+V, where HJJ¯ (on HJJ¯) is the local Hamiltonian of the *J*-th subsystem, $I_{\bar{J}}$ is the identity on the direct product $H_{\bar{J}}={⨂}_{K\neq J}H_{K}$ of all the other subspaces, and *V* (on H) is the interaction Hamiltonian.

The linear von Neumann law of evolution, $\dot{\rho }=-i\left[H,\rho \right]/\hbar$, has a universal structure and involves local evolutions through partial tracing,

(13) ρ˙JJ¯=−iℏ[HJJ¯,ρJJ¯]−iℏTrJ¯([V,ρ]).

Thus, we recover the universal law ρ˙JJ¯=−i[HJJ¯,ρJJ¯]/ℏ for the local density operator ρJJ¯=TrJ¯(ρ) if the subsystem JJ¯ does not interact with the others (i.e., if V=IJJ¯⊗VJ¯).

Instead, a fully nonlinear evolution equation for the density operator cannot have a universal structure because the subdivision into subsystems must be explicitly embedded into the structure of the dynamical law (see [24] for more on this). A different subdivision requires a different equation of motion. The complex structure of the SEA evolution law reflects the cost of abandoning linearity but ensures compatibility with the crucial constraint that correlations should not build up, and signaling should not occur between subsystems, other than via the interaction Hamiltonian *V* through the standard Schrödinger term −i[H,ρ]/ℏ in the evolution law.

Seldom used in composite quantum dynamics but crucial, in our opinion, are the physical observables first introduced in [17] and later (starting with [19]) referred to as ‘local perceptions of global observables’. These are represented via the ‘local perception operators’ (LPO) on HJJ¯, defined along with their ‘deviation from the local mean value’ operators and covariance functionals as follows:

(14) (X)ρJ=TrJ¯[(IJJ¯⊗ρJ¯)X],

(15) Δ(X)ρJJ¯=(X)ρJJ¯−IJJ¯Tr[ρJJ¯(X)ρJJ¯],

(16) (X,Y)ρJJ¯=12Tr[ρJJ¯Δ(X)ρJJ¯,Δ(Y)ρJJ¯],

where ρJ¯=TrJJ¯(ρ). For a bipartite system, AB, the LPOs ${\left(X\right)}_{\rho }^{A}$ (on $H_{A}$) and ${\left(X\right)}_{\rho }^{B}$ (on $H_{B}$) are the unique operators that, for a given *X* on $H_{AB}$ and for all states ρ, satisfy the identity

(17) $$\mathrm{Tr}\left[\rho_{A}{\left(X\right)}_{\rho }^{A}\right]=\mathrm{Tr}\left[\left(\rho_{A}⊗\rho_{B}\right)X\right]=\mathrm{Tr}\left[\rho_{B}{\left(X\right)}_{\rho }^{B}\right]\;.$$

This confirms that they encapsulate all the information that *A* and *B* can infer about the global observable X by classically sharing their local states.

Operator ${\left(X\right)}_{\rho }^{A}$ can be viewed as the projection onto $H_{A}$ of the operator *X* weighted according to the local state $\rho_{B}$ of subsystem *B*. However, it is a local observable for subsystem A, which depends on the overall state, ρ, and the overall observable, *X*. Its local mean value, $\mathrm{Tr}[\rho_{A}{\left(X\right)}_{\rho }^{A}]$, differs from the mean value, Tr(ρX), for the overall system, AB, except when *A* and *B* are uncorrelated ($\rho =\rho_{A}⊗\rho_{B}$). It was dubbed ‘local perception’ because, even if *B* performs a local tomography and sends the measured $\rho_{B}$ to *A* via classical communication, the most that *A* can measure locally about the overall observable *X* is ${\left(X\right)}_{\rho }^{A}$.

The overall energy and entropy of the composite system are locally perceived within subsystem JJ¯ through the operators (H)ρJJ¯ and (S)ρJJ¯ defined on HJJ¯ by Equation (14), respectively, with X=H, the overall Hamiltonian, and X=S(ρ), the overall (non-negative) entropy operator defined by

(18) $$S\left(\rho \right)=-k_{B}\mathrm{Bln}\left(\rho \right)\;,$$

where we define the discontinuous log function

(19) $$\mathrm{Bln}\left(x\right)\;=\;\left\{\begin{array}{c} \ln(x),\;\;\mathrm{for}\;0<x\leq 1,\\ 0,\;\;\mathrm{otherwise}. \end{array}\right.$$

Note that the ‘locally perceived overall entropy’ operator

(20) (S)ρJ=−kBTrJ¯[(IJJ¯⊗ρJ¯)Bln(ρ)],

is different from the ‘local entropy’ operator

(21) S(ρJJ¯)=−kBBln(ρJJ¯).

Its local mean value, Tr[ρJJ¯(S)ρJJ¯]=−kBTr[(ρJJ¯⊗ρJ¯)Bln(ρ)], is different from the local entropy Tr[ρJJ¯S(ρJJ¯)]=−kBTr[ρJJ¯ln(ρJJ¯)]. Only when ρ=ρJJ¯⊗ρJ¯ are they related via

(22) Tr[ρJJ¯(S)ρJJ¯]=Tr[ρJJ¯S(ρJJ¯)]+Tr[ρJ¯S(ρJ¯)]=−kBTr[ρln(ρ)].

Likewise, the ‘locally perceived overall Hamiltonian’ operator (H)ρJJ¯ differs from the ‘local Hamiltonian’ operator HJJ¯. Its local mean value, Tr[ρJJ¯(H)ρJJ¯]=Tr[(ρJJ¯⊗ρJ¯)H], is different from the local mean energy, Tr(ρJJ¯HJJ¯), and only when V=IJJ¯⊗VJ¯ are they related via

(23) Tr[ρJJ¯(H)ρJJ¯]=Tr(ρJJ¯HJJ¯)+Tr(ρJ¯HJ¯)=Tr(ρH).

However, it is noteworthy that, when the overall observable, *X* is ‘separable for subsystem JJ¯,’ in the sense that X=XJJ¯⊗IJ¯+IJJ¯⊗XJ¯, then, even if ρ≠ρJJ¯⊗ρJ¯, the deviations and covariances reduce to their local versions,

(24) Δ(X)ρJJ¯=ΔXJJ¯=XJJ¯−IJJ¯Tr[ρJJ¯XJJ¯],

(25) (X,Y)ρJJ¯=12Tr[ρJJ¯ΔXJJ¯,ΔYJJ¯].

In general, the mean interaction energy, Tr(ρJJ¯VJJ¯J¯), and the mutual information, Tr(ρJJ¯μJJ¯J¯), between subsystem JJ¯ and the rest of the system, $\bar{J}$, are given via the respective mean values of the following global operators:

(26) VJJ¯J¯=H−HJJ¯⊗IJ¯−IJJ¯⊗HJ¯,

(27) μJJ¯J¯=Bln(ρ)−Bln(ρJJ¯)⊗IJ¯−IJJ¯⊗Bln(ρJ¯),

whose LPOs satisfy the identities

(28) Tr[ρJJ¯(H)ρJJ¯]−Tr[ρJJ¯(VJJ¯J¯)ρJJ¯]=Tr(ρJJ¯HJJ¯)+Tr(ρJ¯HJ¯)=Tr(ρH)−Tr(ρJJ¯VJJ¯J¯),

(29) Tr[ρJJ¯(S)ρJJ¯]+kBTr[ρJJ¯(μJJ¯J¯)ρJJ¯]=−kBTr[ρJJ¯ln(ρJJ¯)]−kBTr[ρJ¯ln(ρJ¯)]=−kBTr[ρln(ρ)]+kBTr(ρJJ¯μJJ¯J¯).

## 7. No-Signaling and LPOs

To formalize the no-signaling definition following [22], as discussed above, we adopt the view that local operations can be acted on a subsystem, $\bar{J}$, only by means of a controlled time dependence of its local Hamiltonian operator, $H_{\bar{J}}$, or its interaction operator, $V_{\bar{J}B}$, with some other subsystem, *B* (possibly a properly modeled environment or heat bath). Hence, we adopt the following:

**Definition 1**  (No-signaling). *If JJ¯ and $\bar{J}$ are non-interacting, no local aspect of the time evolution of JJ¯ can be affected by local unitary operations acted on $\bar{J}$, nor by other aspects of the local time evolution of $\bar{J}$.*

Accordingly, consider a composite, AB, in state ρ. A local unitary operation on *B* changes the state to

(30) $$\rho^{\prime }=\left(I_{A}⊗U_{B}\right)\;\rho \;\left(I_{A}⊗U_{B}^{†}\right)\;,$$

where $U_{B}$ is an arbitrary unitary operator ($U_{B}^{†}U_{B}=I_{B}$). Using the properties of the partial trace, in particular ($Z_{AB}$ is a generic composite system operator),

(31) $$\begin{array}{cc} \; & {\mathrm{Tr}}_{B}\left[\left(I_{A}⊗X_{B}\right)Z_{AB}\right]={\mathrm{Tr}}_{B}\left[Z_{AB}\left(I_{A}⊗X_{B}\right)\right]\;, \end{array}$$

(32) $$\begin{array}{cc} \; & {\mathrm{Tr}}_{A}\left[\left(I_{A}⊗X_{B}\right)Z_{AB}\left(I_{A}⊗Y_{B}\right)\right]=X_{B}{\mathrm{Tr}}_{A}\left(Z_{AB}\right)Y_{B}\;, \end{array}$$

we obtain the identities

(33) $$\begin{array}{cc} \rho_{B} & ={\mathrm{Tr}}_{A}\left[\left(I_{A}⊗U_{B}^{†}\right)\;\rho^{\prime }\;\left(I_{A}⊗U_{B}\right)\right]=U_{B}^{†}\rho_{B}^{\prime }U_{B}, \end{array}$$

(34) $$\begin{array}{cc} \rho_{A}^{\prime } & ={\mathrm{Tr}}_{B}\left[\left(I_{A}⊗U_{B}\right)\;\rho \;\left(I_{A}⊗U_{B}^{†}\right)\right]\\ \; & ={\mathrm{Tr}}_{B}\left[\left(I_{A}⊗U_{B}^{†}U_{B}\right)\;\rho \right]\\ \; & ={\mathrm{Tr}}_{B}\left[\left(I_{A}⊗I_{B}\right)\;\rho \right]=\rho_{A}, \end{array}$$

which confirms that a local operation on *B* does not affect the local state, $\rho_{A}$, of *A*. This result supports the usual idea [46] that, for no-signaling, it is sufficient that the dynamical model implies evolutions of local observables that depend only on $\rho_{A}$. But it is seldom noted that this is not a necessary condition.

Next, we prove that not only the local reduced state $\rho_{A}$ but also the LPO ${\left(F\left(\rho \right)\right)}^{A}$ of any well-defined nonlinear function, F(ρ), of the overall state (such as the entropy function S(ρ) defined earlier) remains unaffected by local unitary operations on *B*, as per Equation (30). Since the SEA formalism is based on these local perception operators, this result is an important lemma in the proof that SEA is no-signaling.

So, let us apply Equation (30) to a generic observable represented by a linear operator on H that we denote as F(ρ), whether it is a nontrivial function of ρ, such as S(ρ), or not a function of ρ, such as *H*. The corresponding LPO for subsystem A, according to the defining Equation (14), is given by

(35) $${\left(F\left(\rho \right)\right)}^{A}\;=\;{\mathrm{Tr}}_{B}\left[\left(I_{A}⊗\rho_{B}\right)F\left(\rho \right)\right].$$

A function of ρ is defined from its eigen-decomposition via

(36) $$F\left(\rho \right)\;=\;\Lambda F\left(D\right)\Lambda^{†}=\sum_{j}F\left(\lambda_{j}\right)|\lambda_{j}\rangle \langle \lambda_{j}|,$$

where $\rho =\Lambda D\Lambda^{†}$, $D=\sum_{j}\lambda_{j}\left|j\rangle \langle j\right|$, and $\Lambda =\sum_{j}|\lambda_{j}\rangle \langle j|$. Since unitary transformations do not alter the eigenvalues,

(37) $$F\left(\rho^{\prime }\right)\;=\;\Lambda^{\prime }F\left(D\right)\Lambda^{\prime †}\;\mathrm{where}\;\Lambda^{\prime }=\left(I_{A}⊗U_{B}\right)\Lambda \;.$$

Therefore, using Equation (33) in the last step, we obtain

(38) $$\begin{array}{cc} \; & {\left(F\left(\rho^{\prime }\right)\right)}^{A}\;=\;{\mathrm{Tr}}_{B}\left[\left(I_{A}⊗\rho_{B}^{\prime }\right)F\left(\rho^{\prime }\right)\right]\\ & ={\mathrm{Tr}}_{B}\left[\left(I_{A}⊗\rho_{B}^{\prime }\right)\;\left(I_{A}⊗U_{B}\right)\Lambda F\left(D\right)\Lambda^{†}\left(I_{A}⊗U_{B}^{†}\right)\right]\\ & ={\mathrm{Tr}}_{B}\left[\left(I_{A}⊗U_{B}^{†}\rho_{B}^{\prime }U_{B}\right)\;\Lambda F\left(D\right)\Lambda^{†}\right]\\ \; & ={\mathrm{Tr}}_{B}\left[\left(I_{A}⊗\rho_{B}\right)\;F\left(\rho \right)\right]={\left(F\left(\rho \right)\right)}^{A}\;. \end{array}$$

This confirms that local unitary operations on *B* do not affect the LPOs of *A*. Hence, the proper use of LPOs in a nonlinear evolution equation does not cause signaling issues.

## 8. The Local Structure of Dissipation in Composite-System SEA Dynamics

We are now ready to introduce the final but essential ingredient of a general composite-system nonlinear QM or master equation. This involves the system’s structure-dependent expressions, which determine how each subsystem contributes separately to the dissipative term in the equation of motion for the overall state, ρ. As discussed above—and recognized in early SEA literature [17,22,24]—the composite-system nonlinear evolution must explicitly reflect the internal structure of the system. This requires declaring which subsystems are to be protected from nonphysical effects such as signaling, the exchange of energy, or the build-up of correlations between non-interacting subsystems. Using the notation introduced earlier, the structure proposed in [17,24] for the dissipative term, which supplements the usual Hamiltonian term, is given by

(39) dρdt=−iℏH,ρ−∑JJ¯=1MDρJJ¯,ρJJ¯⊗ρJ¯,

where the ‘local dissipation operators’ DρJJ¯ (on HJJ¯) may be nonlinear functions of the local observables of JJ¯, the reduced state ρJJ¯, and the local perception operators of overall observables. For the dissipative term to preserve Tr(ρ), operators DρJJ¯,ρJJ¯ must be traceless. To preserve Tr(ρH) [and possibly other conserved properties or charges, $\mathrm{Tr}(\rho C_{k})$], operators DρJJ¯,ρJJ¯(H)ρJJ¯ [and DρJJ¯,ρJJ¯(Ck)ρJJ¯] must also be traceless. The rate of change of the overall system entropy $s\left(\rho \right)=-k_{B}\mathrm{Tr}\left[\rho \ln\left(\rho \right)\right]$ is

(40) ds(ρ)dt=−∑JJ¯=1MTr[DρJJ¯,ρJJ¯(S)ρJJ¯].

The local nonlinear evolution of subsystem JJ¯ is obtained via partial tracing over $H_{\bar{J}}$, i.e., in general,

(41) dρJJ¯dt=−iℏHJJ¯,ρJJ¯−iℏTrJ¯(V,ρ)−DρJJ¯,ρJJ¯,

where we recall that the second term in the RHS can be expressed, for weak interactions and under well-known assumptions, in Kossakowski–Lindblad form.

Before introducing the SEA assumption, as promised after Equation (2), we note that, for all possible choices of DρJJ¯, Equation (39) defines a broad class of no-signaling nonlinear evolution equations. These form a broader class of nonlinear laws that are not restricted by the sufficient but not necessary condition that dρJJ¯/dt be a function of ρJJ¯ only.

Finally, one way to introduce the SEA assumption in the spirit of the fourth law of thermodynamics [65,66,67,68], i.e., to implement the maximum entropy production principle (MEPP) in the present context [69], is to employ a variational principle.

## 9. General Composite System Version of the SEA Variational Principle

For the purpose of this section, we adopt the formalism developed for this context in [17,20,24]. To easily impose the constraints of preservation of non-negativity and self-adjointness of ρ during its time evolution, we define the generalized square root of ρJJ¯, γJJ¯(t)=ρJJ¯(t)U (*U* any unitary operator), so that

(42) ρJJ¯=γJJ¯γJJ¯†.

The dissipative term in Equation (41) is rewritten as

(43) −DρJJ¯,ρJJ¯=γ˙JJ¯dγJJ¯†+γJJ¯γ˙JJ¯d†

(44) withγ˙JJ¯d=−DρJJ¯γJJ¯.

Next, on the set L(HJJ¯) of linear operators on HJJ¯, we define the real inner product, (·|·),

(45) $$\left(X|Y\right)=\mathrm{Tr}\left(X^{†}Y+Y^{†}X\right)/2\;,$$

so that the unit trace condition for ρJJ¯ is rewritten as (γJJ¯|γJJ¯)=1, implying that the γJJ¯’s lie on the unit sphere in L(HJJ¯). Their time evolutions, γJJ¯(t), follow trajectories on this sphere. Along these trajectories, we can express the distance traveled between *t* and t+dt as

(46) dlJJ¯=(γ˙JJ¯|G^JJ¯(γJJ¯)|γ˙JJ¯)dt,

where G^JJ¯(γJJ¯) is some real, dimensionless, symmetric, and positive–definite operator on L(HJJ¯) (superoperator on H) that plays the role of a local metric tensor field (and may be a nonlinear function of γJJ¯).

The rates of change of the overall system entropy, s(ρ), Equation (40), and of the overall system mean value of conserved properties $c_{k}\left(\rho \right)=\mathrm{Tr}\left(\rho C_{k}\right)$, where $\left[C_{k},H\right]=0$, can be rewritten as

(47) ds(ρ)dt=∑JJ¯=1Ms˙|JJ¯s˙|JJ¯=2(S)ρJJ¯γJJ¯|γ˙JJ¯d,

(48) dck(ρ)dt=∑JJ¯=1Mck˙|JJ¯ck˙|JJ¯=2(Ck)ρJJ¯γJJ¯|γ˙JJ¯d,

exhibiting additive contributions from the subsystems.

Finally, we state the variational principle that leads to expressions for the γ˙JJ¯d and the DρJJ¯ that define the composite-system version of the SEA equation of motion. The time evolution ensures that the ’direction of change’ of the local trajectory γJJ¯(t), influenced by the dissipative part of the dynamics, maximizes the local contribution, s˙|JJ¯, to the overall system’s entropy production rate. This occurs under the constraints ck˙|JJ¯=0 that guarantee no local contribution to the rates of change of the global constants of the motion. With the introduction of Lagrange multipliers ϑkJJ¯ and τJJ¯ for the constraints, the γ˙JJ¯d’s are found by solving the maximization problem

(49) maxγ˙JJ¯dΥJJ¯=s˙|JJ¯−∑kϑkJJ¯ck˙|JJ¯−kBτJJ¯2γ˙JJ¯d|G^JJ¯|γ˙JJ¯d,

where the last constraint corresponds to the condition (dℓJJ¯d/dt)2=const, necessary for maximizing with respect to direction only (see [69] for more details). Taking the variational derivative of ΥJJ¯ with respect to |γ˙JJ¯d) and setting it equal to zero, we obtain

(50) δΥJJ¯|δγ˙JJ¯d)=|2(M)ρJJ¯γJJ¯)−kBτJJ¯G^JJ¯|γ˙JJ¯d)=0,

where we used the identity (X|G^JJ¯=G^JJ¯|X), following from the symmetry of G^JJ¯. We define (following [23,70]) the ’locally perceived non-equilibrium Massieu operator’

(51) (M)ρJJ¯=(S)ρJJ¯−∑kϑkJJ¯(Ck)ρJJ¯.

Equation (50) yields

(52) |γ˙JJ¯d)=1kBτJJ¯G^JJ¯−1|2(M)ρJJ¯γJJ¯),

where the Lagrange multipliers ϑkJJ¯ (implicit in (M)ρJJ¯) are the solution of the system of equations, obtained by substituting Equation (52) into the conservation constraints,

(53) (Cℓ)ρJJ¯γJJ¯|G^JJ¯−1|(M)ρJJ¯γJJ¯=0∀ℓ.

This system can be solved explicitly for the ϑkJJ¯’s using Cramer’s rule, to obtain convenient expressions for the γ˙JJ¯d’s as ratios of determinants (as in the original formulations). The ϑkJJ¯’s are nonlinear functionals of ρ that may be interpreted as ’local non-equilibrium entropic potentials’ conjugated with the conserved properties. For example, for $C_{2}=H$, the Lagrange multiplier ϑ2JJ¯ plays the role of ’local non-equilibrium inverse temperature’ conjugated with the locally perceived energy, and for the stable equilibrium states of the SEA dynamics, it coincides with the thermodynamic inverse temperature kBβJJ¯ (see below).

Similarly to what was achieved in [69] for a non-composite system, we define the ’local non-equilibrium affinity’ operators

(54) |ΛJJ¯)=G^JJ¯−1/2|2(M)ρJJ¯γJJ¯),

so that the overall rate of entropy production becomes

(55) ds(ρ)dt=∑JJ¯=1M(ΛJJ¯|ΛJJ¯)kBτJJ¯.

(ΛJJ¯|ΛJJ¯) is the norm of 2(M)ρJJ¯γJJ¯ with respect to the metric G^JJ¯−1 and may be interpreted as the ‘degree of disequilibrium’ of subsystem JJ¯. Hence, the necessary and sufficient condition for the overall state to be locally non-dissipative (no contribution to the overall entropy production from subsystem JJ¯) is that operator 2(M)ρJJ¯γJJ¯ vanishes.

However, in order for the equation of motion (41) to result independent of the unitary operators *U* used (in γJJ¯=ρJJ¯U) to define the generalized square roots of ρJJ¯, we further restrict the choice of the metric superoperator G^JJ¯. We assume that G^JJ¯=LJJ¯−1I^JJ¯, with LJJ¯ being some strictly positive, hermitian operator on HJJ¯, possibly a nonlinear function of ρJJ¯, so that G^JJ¯|X)=|LJJ¯−1X), G^J−1|X)=|LJJ¯X),

(56) (XγJJ¯|G^J−1|YγJJ¯)=12TrρJJ¯(X†LJJ¯Y+Y†LJJ¯X).

With the recollection of Equations (43) and (44), and using Equation (52), the dissipative term in Equation (41) becomes

(57) −DρJJ¯,ρJJ¯=2kBτJJ¯LJJ¯(M)ρJJ¯ρJJ¯+ρJJ¯(M)ρJJ¯LJJ¯,

and the system of equations that determines the Lagrange multipliers ϑkJJ¯ in (M)ρJJ¯ is

(58) TrρJJ¯(Cℓ)ρJJ¯LJJ¯(M)ρJJ¯+(M)ρJJ¯LJJ¯(Cℓ)ρJJ¯=0∀ℓ

so that the dependence on γJJ¯ is only through the product γJJ¯γJJ¯†, i.e., the local state operator ρJJ¯.

The metric operator LJJ¯−1/kBτJJ¯ plays a role analogous to the symmetric thermal conductivity tensor $\hat{k}$ in heat transfer theory. In that context, $\hat{k}$ defines the general near-equilibrium linear relationship, $|q^{\prime \prime })=-\hat{k}\;\left|\nabla T\right)$, between the heat flux vector $q^{\prime \prime }$ and the conjugated ’degree of disequilibrium’ vector, i.e., the temperature gradient ∇T. Here, Equation (57) expresses a more general linear relationship between the local evolution operator γ˙JJ¯d and the non-equilibrium Massieu operator, (S)ρJJ¯−∑kϑkJJ¯(Ck)ρJJ¯. This relation is more general, as it holds not only near equilibrium but also anywhere far from equilibrium. In the present quantum modeling context, it represents the nonlinear SEA extension into the far-non-equilibrium domain of Onsager’s linear near-equilibrium theory, with reciprocity naturally embedded through the symmetry of any metric. For example, if LJJ¯ commutes with the local Hamiltonian HJJ¯, its eigenvalues can assign different relaxation times to the local energy levels, capturing their uneven contributions to the rate of local energy redistribution described by the SEA dissipator DρJJ¯.

Like in heat transfer, the conductivity tensor for an isotropic material is $\hat{k}=k\;\hat{I}$; here, an analogous simplification is obtained when LJJ¯=IJJ¯, the identity operator on HJJ¯. This corresponds to assuming a uniform Fisher–Rao metric.

Another noteworthy observation is that operators $C_{k}$, to be global constants of the motion (or ’charges’), must commute with the global Hamiltonian operator *H*, but they need not commute with each other. Therefore, the SEA formalism may also find application in the framework of quantum thermodynamic resource theories that contemplate ’non-commuting charges’ and ’non-Abelian thermal states’, as discussed in Yunger Halpern et al. [11] and Murthy et al. [71].

## 10. Simplest Composite-System SEA Equation of Motion

For simplicity, we proceed by assuming the following: (1) a uniform Fisher–Rao metric, with G^JJ¯=I^JJ¯, so that G^JJ¯|X)=|X) and LJJ¯=IJJ¯; and (2) only two global conserved properties, $c_{1}\left(\rho \right)=\mathrm{Tr}\left(\rho I\right)$ (the normalization condition) and $c_{2}\left(\rho \right)=\mathrm{Tr}\left(\rho H\right)$ (mean energy conservation).

The first assumption allows the obtaining of

(59) DρJJ¯=−2kBτJJ¯(S)ρJJ¯−∑kϑkJJ¯(Ck)ρJJ¯.

and the system of Equation (58) reduces to

(60) ∑kϑkJJ¯Tr[ρJJ¯(Cℓ)ρJJ¯,(Ck)ρJJ¯]=Tr[ρJJ¯(S)ρJJ¯,(Cℓ)ρJJ¯].

The second assumption, $C_{1}=I$ and $C_{2}=H$, together with definitions (15) and (16), allows the writing of the SEA dissipators in the compact forms

(61) −DρJ,ρJ=2kBτJJ¯Δ(M)ρJJ¯,ρJ=2kBτJJ¯Δ(S)ρJJ¯,ρJΔ(H)ρJJ¯,ρJ(H,S)ρJJ¯(H,H)ρJJ¯(H,H)ρJJ¯,

and the local non-equilibrium inverse temperatures conjugated with the locally perceived energy as

(62) ϑ2JJ¯=(H,S)ρJJ¯(H,H)ρJJ¯.

As a result, the local evolution of each subsystem, JJ¯, is along the direction of steepest ascent of the locally perceived overall composite-system entropy, compatible with the conservation of the locally perceived overall composite-system energy and the conditions of unit-trace and non-negativity of the overall density operator, ρ. The rate of entropy production may be expressed as

(63) ds(ρ)dt=∑J=1M4kBτJJ¯(M,M)ρJJ¯=∑J=1M4kBτJJ¯(S,S)ρJJ¯(H,S)ρJJ¯(H,S)ρJJ¯(H,H)ρJJ¯(H,H)ρJJ¯,

showing clearly that it is non-negative since the numerators in the summation are Gram determinants. When the JJ¯-th term in the sum vanishes, we say that the local state ρJJ¯ is ‘non-dissipative’. If all local states are non-dissipative, then so is the overall state ρ. A non-dissipative ρ represents an equilibrium state if it commutes with *H*; otherwise, it belongs to a unitary limit cycle of the dynamics.

For ρ to be non-dissipative, there must exist $\beta_{J}$s, such that, for every JJ¯,

(64) ρJJ¯Δ(Bln(ρ))ρJJ¯=−βJρJJ¯Δ(H)ρJJ¯,

or, equivalently,

(65) ρJJ¯ΔBln(ρJJ¯)+βJHJJ¯+βJ(VJJ¯J¯)ρJJ¯+(μJJ¯J¯)ρJJ¯=0,

where VJJ¯J¯ is the Hamiltonian interaction operator defined in Equation (26), and μJJ¯J¯ the mutual information operator defined in Equation (27). Clearly, for VJJ¯J¯=0 and μJJ¯J¯=0, Equation (65) is satisfied by the Gibbs states

(66) ρJJ¯=exp(−βJJ¯HJJ¯)/Tr[exp(−βJJ¯HJJ¯)].

Section 11.3 provides a numerical illustration of local time evolutions that converge to limit cycles obeying Equation (65). These examples also illustrate another noteworthy possibility. Specifically, without violating the no-signaling condition nor the second-law principle of global entropy non-decrease—which is always guaranteed by virtue of Equation (63) and the properties of Gram determinants—it is possible that the local entropy of a subsystem be decreasing during part of the time evolution. Indeed, for correlated states, the non-decrease in local entropy is not a second-law requirement. We hope that the observation that a local entropy decrease may be thermodynamically consistent in the presence of strong entanglement will stimulate the design of experimental verifications, as well as foundational discussions in the framework of quantum thermodynamic resource theories, even beyond and outside the SEA framework.

Regarding no-signaling, we note the following:

1. If subsystem JJ¯ is non-interacting, VJJ¯J¯=0, then
  (67) Δ(H)ρJJ¯=HJJ¯−IJJ¯Tr(ρJJ¯HJJ¯)=ΔHJJ¯
  and
  (68) (H,H)ρJJ¯=Tr[ρJ(ΔHJJ¯)2]
  depend only on the local HJJ¯ and ρJJ¯;
2. If JJ¯ is uncorrelated, μJJ¯J¯=0, then
  (69) Δ(Bln(ρ))ρJJ¯=Bln(ρJJ¯)−IJJ¯Tr(ρJJ¯ln(ρJJ¯))
  and
  (70) (Bln(ρ),Bln(ρ))ρJJ¯=Tr[ρJJ¯ln(ρJJ¯)2]
  depend only on the local ρJJ¯.

Therefore, it is only when JJ¯ is both non-interacting and uncorrelated in that its local dissipation operator, DρJJ¯, depends only on the local HJJ¯ and ρJJ¯. In this case, the local equation of motion Equation (41), along with DρJJ¯ given by Equation (61), reduces exactly to the non-composite system version of SEA evolution [16]. Instead, if JJ¯ is either interacting or correlated, DρJJ¯ and, therefore, the local nonlinear SEA evolution, according to Equations (41) and (61), is determined not only by the local HJJ¯ and ρJJ¯ but also by the local perceptions of the overall Hamiltonian operator *H* and/or the overall entropy operator Bln(ρ), nonetheless without violating the no-signaling condition.

To prove no-signaling, assume that subsystem JJ¯ is correlated but not interacting with any of the subsystems in $\bar{J}$. Now, switch on an arbitrary interaction that may involve the subsystems in $\bar{J}$ but not subsystem JJ¯, so that the only change in the overall Hamiltonian *H* is the term $H_{\bar{J}}$, which changes to $H_{\bar{J}}^{\prime }$. Within JJ¯, the locally perceived deviation operators are Δ(H)ρJJ¯ and Δ(Bln(ρ))ρJJ¯, and hence, the SEA dissipator DρJJ¯, as well as all the terms in the RHS of Equation (41), are not modified by such a change. Therefore, acting within $\bar{J}$ makes it impossible to affect the time evolution of ρJJ¯ and any local observable of JJ¯.

We also mention the following in the spirit of non-linear evolutions. In the case of a general open quantum dynamics, the requirement that a map, $W_{t}\left(\rho \right)$, be completely positive and trace-preserving (CPTP) is quite restrictive [72,73,74]. In fact, the reduced dynamics of the subsystem in interaction with the environment need not be CPTP [75,76,77]. Here, we define CPTP as follows. If $M(\rho_{s})$ is a map acting on a subsystem in state $\rho_{s}$, which interacts with an initially uncorrelated environment in state $\rho_{E}$ and is positive and trace-preserving—meaning it preserves the trace and ensures that the evolved matrix remains semi-definite (maintaining probability conservation)—then $M(\rho_{s})$ is CPTP if and only if the extended map, $\Lambda :M\left(\rho_{s}\right)⊗I_{N}$, remains positive for all *N*s [72]. As has been argued in the literature, the CPTP condition is restrictive because, along with the underlying assumption of Markovianity, it requires a preparation of the initial state into a product state of system and environment. But, if there are strong initial correlations between system and environment, or in fact, if the evolution is non-Markovian in nature, then this requirement fails, and we are forced to consider PTP (positive and trace-preserving) maps only [78,79]. Given that we are dealing with a theory that does not restrict itself to Markovian evolution, and that there is no imposition of weak interaction between the subsystems, SEA being PTP satisfies the requirements for a suitable nonlinear model of quantum thermodynamics prohibiting signaling.

## 11. Non-Interacting Qubits

In extreme cases, Equation (65) shows that, even if the subsystems are entangled, and therefore, the local states ρJJ¯ are mixed, operators DρJJ¯ may vanish. Equations (39) and (41) reduce to the standard Schrödinger equation, and the trajectory in state space is a limit cycle of the SEA dynamics. A noteworthy particular case is when the overall system is in a pure state. Then, Bln(ρ)=0 and standard unitary evolutions of pure states emerge as limit cycles of the nonlinear SEA dynamics. In this section, we discuss a few less trivial examples, to numerically illustrate some general features of Equation (39) with DρJJ¯ given by Equation (61).

We consider examples of mixed and correlated initial states of a two-qubit composite, AB, that belong to the special class

(71) $$\begin{array}{c} \begin{array}{cc} \; & \rho =\frac{1}{4}\left[I_{4}+\;\;\;\;\;\;\sum_{j=\{x,y,z\}}\;\;\;\;\left(a_{j}\;\sigma_{j}⊗I_{2}+b_{j}\;I_{2}⊗\sigma_{j}+c_{j}\;\sigma_{j}⊗\sigma_{j}\right)\right]\\ \; & =\frac{1}{4}\;\;\left(\;\begin{array}{cccc} 1+a_{z}+b_{z}+c_{z}\;\;\;\;\; & b_{x}-ib_{y} & a_{x}-ia_{y} & c_{x}-c_{y}\\ b_{x}+ib_{y} & \;\;\;\;\;1+a_{z}-b_{z}-c_{z}\;\;\;\;\; & c_{x}+c_{y} & a_{x}-ia_{y}\\ a_{x}+ia_{y} & c_{x}+c_{y} & \;\;\;\;\;1-a_{z}+b_{z}-c_{z}\;\;\;\;\; & b_{x}-ib_{y}\\ c_{x}-c_{y} & a_{x}+ia_{y} & b_{x}+ib_{y} & \;\;\;\;\;1-a_{z}-b_{z}+c_{z} \end{array}\right), \end{array} \end{array}$$

with real $a_{j}$’s, $b_{j}$’s, $c_{j}$’s, such that $a^{2}=a\cdot a\leq 1$, $b^{2}=b\cdot b\leq 1$, $c^{2}=c\cdot c\leq 3-a^{2}-b^{2}$, plus other conditions necessary for non-negativity (see e.g., [80,81,82]. We will denote the eigenvalues of ρ as $\lambda_{j}$ and, for shorthand, define

(72) $$\eta_{j}=-k_{B}\mathrm{Bln}\left(\lambda_{j}\right)\;.$$

We further assume that the two qubits are non-interacting and have local Hamiltonian operators given by HJJ¯=ωJJ¯hJJ¯·σJJ¯, where σJJ¯ denotes the 3-vector formed by the local Pauli operators of subsystem JJ¯, and hJJ¯ is the local Hamiltonian unit 3-vector, so that

(73) H=HA⊗I2+I2⊗HBH=ωAhA·σA⊗I2+I2⊗ωBhB·σB,(H)ρA=HA+I2ωBhB·b,(H)ρB=I2ωAhA·a+HBΔ(H)A=ΔHA,Δ(H)B=ΔHB,(H,H)ρA=[1−(hA·a)2]ωA2,(H,H)ρB=[1−(hB·b)2]ωB2,

### 11.1. Bell Diagonal States

Equation (71) gives Bell diagonal states [83,84] (BDS) if $a_{j}=b_{j}=0$ for all *j*s (and Werner states [85] if, in addition, $c_{j}=4w/3-1$ for all *j*s),

(74) $$\begin{array}{cc} \; & \rho^{\mathrm{Bell}}=\frac{1}{4}\left(\begin{array}{cccc} 1+c_{z} & 0 & 0 & c_{x}-c_{y}\\ 0 & 1-c_{z} & c_{x}+c_{y} & 0\\ 0 & c_{x}+c_{y} & 1-c_{z} & 0\\ c_{x}-c_{y} & 0 & 0 & 1+c_{z} \end{array}\right)=\frac{1}{2}\left(\begin{array}{cccc} \lambda_{2}+\lambda_{3} & 0 & 0 & \lambda_{3}-\lambda_{2}\\ 0 & \lambda_{1}+\lambda_{4} & \lambda_{4}-\lambda_{1} & 0\\ 0 & \lambda_{4}-\lambda_{1} & \lambda_{1}+\lambda_{4} & 0\\ \lambda_{3}-\lambda_{2} & 0 & 0 & \lambda_{2}+\lambda_{3} \end{array}\right),\\ \; & \;\;=\rho_{A}⊗\rho_{B}+\sum_{j=\{x,y,z\}}c_{j}\;\sigma_{j}⊗\sigma_{j}\; \end{array}$$

whose eigen-decomposition may be written as

(75) $$\begin{array}{c} \begin{array}{cc} \; & \rho^{\mathrm{Bell}}\;=\;U^{\mathrm{Bell}}\mathrm{diag}\left(\lambda_{1},\lambda_{2},\lambda_{3},\lambda_{4}\right){\left(U^{\mathrm{Bell}}\right)}^{†},\\ \; & U^{\mathrm{Bell}}\;=\;\frac{\sigma_{x}⊗I_{2}-\sigma_{z}⊗\sigma_{x}}{\sqrt{2}}=\frac{1}{\sqrt{2}}\left(\begin{array}{cccc} 0 & -1 & 1 & 0\\ -1 & 0 & 0 & 1\\ 1 & 0 & 0 & 1\\ 0 & 1 & 1 & 0 \end{array}\right)\;,\\ \; & \lambda_{1}\;=\;\frac{1}{4}\left(1-c_{x}-c_{y}-c_{z}\right)\;,\;\lambda_{2}\;=\;\frac{1}{4}\left(1-c_{x}+c_{y}+c_{z}\right)\;,\\ \; & \lambda_{3}\;=\;\frac{1}{4}\left(1+c_{x}-c_{y}+c_{z}\right)\;,\;\lambda_{4}\;=\;\frac{1}{4}\left(1+c_{x}+c_{y}-c_{z}\right)\;. \end{array} \end{array}$$

The overall entropy operator, Equation (18), becomes

(76) $$\begin{array}{c} \begin{array}{cc} \; & S\left(\rho^{\mathrm{Bell}}\right)\;=\;U^{\mathrm{Bell}}\;\left[\mathrm{diag}\left(\eta_{1},\eta_{2},\eta_{3},\eta_{4}\right)\right]{\left(U^{\mathrm{Bell}}\right)}^{†}\\ \; & \;=\frac{1}{2}\left(\begin{array}{cccc} \eta_{2}+\eta_{3} & 0 & 0 & \eta_{3}-\eta_{2}\\ 0 & \eta_{1}+\eta_{4} & \eta_{4}-\eta_{1} & 0\\ 0 & \eta_{4}-\eta_{1} & \eta_{1}+\eta_{4} & 0\\ \eta_{3}-\eta_{2} & 0 & 0 & \eta_{2}+\eta_{3} \end{array}\right), \end{array} \end{array}$$

and the local operators of the SEA formalism

(77) $$\begin{array}{c} \begin{array}{cc} & \rho_{A}=\rho_{B}=\frac{1}{2}I_{2}\;,\\ \; & {\left(\mathrm{Bln}\left(\rho \right)\right)}^{A}={\left(\mathrm{Bln}\left(\rho \right)\right)}^{B}=-\frac{q}{4k_{B}}I_{2}\;,\\ \; & \left\{{\left(\mathrm{Bln}\left(\rho \right)\right)}^{A},\rho_{A}\right\}=\left\{{\left(\mathrm{Bln}\left(\rho \right)\right)}^{B},\rho_{B}\right\}=-\frac{q}{4k_{B}}I_{2}\;,\\ \; & \left\{\Delta {\left(\mathrm{Bln}\left(\rho \right)\right)}^{A},\rho_{A}\right\}=\left\{\Delta {\left(\mathrm{Bln}\left(\rho \right)\right)}^{B},\rho_{B}\right\}=0\;,\\ \; & {\left(H,H\right)}_{\rho }^{A}\;=\;\omega_{A}^{2}\;,\;{\left(H,H\right)}_{\rho }^{B}=\omega_{B}^{2}\;,\\ \; & {\left(H,\mathrm{Bln}\left(\rho \right)\right)}_{\rho }^{A}\;=\;{\left(H,\mathrm{Bln}\left(\rho \right)\right)}_{\rho }^{B}=0\;. \end{array} \end{array}$$

where *q* is defined as

(78) $$q=\eta_{1}+\eta_{2}+\eta_{3}+\eta_{4}\;.$$

Therefore, somewhat surprisingly, we find that

(79) $$-\left\{D_{\rho }^{A},\rho_{A}\right\}=-\left\{D_{\rho }^{B},\rho_{B}\right\}=0\;,$$

i.e., the Bell diagonal states are non-dissipative limit cycles of the nonlinear SEA dynamics under any Hamiltonian. But most neighboring and other states in the class defined by Equation (71) are dissipative, as we see in the following examples.

### 11.2. Separable, but Correlated Mixed States

For a simple example of correlated but separable mixed states, assume Equation (71) with $a_{x}=a$, $b_{z}=b$, and $a_{y}=a_{z}=b_{x}=b_{y}=c_{x}=c_{y}=c_{z}=0$, so that

(80) $$\begin{array}{c} \begin{array}{cc} \rho & =\frac{1}{4}\left(\;\begin{array}{cccc} 1+b & 0 & a & 0\\ 0 & 1-b & 0 & a\\ a & 0 & 1+b & 0\\ 0 & a & 0 & 1-b \end{array}\right)=\rho_{A}⊗\rho_{B}-\frac{1}{4}ab\;\sigma_{x}⊗\sigma_{z}\;,\\ \lambda_{1} & \;=\;\frac{1}{4}\left(1-a-b\right)\;,\;\lambda_{2}\;=\;\frac{1}{4}\left(1-a+b\right)\;,\\ \lambda_{3} & =\frac{1}{4}\left(1+a-b\right)\;,\;\lambda_{4}\;=\;\frac{1}{4}\left(1+a+b\right). \end{array} \end{array}$$

If the two non-interacting qubits have local Hamiltonians $H_{A}=\sigma_{z}$ and $H_{B}=\sigma_{x}$, we find

(81) $$\begin{array}{cc} -\left\{D_{\rho }^{A},\rho_{A}\right\} & \;=\;\frac{\left(1-a^{2}\right)\left(bf-g\right)}{2k_{B}\tau_{A}}\;\sigma_{x}, \end{array}$$

(82) $$\begin{array}{cc} -\left\{D_{\rho }^{B},\rho_{B}\right\} & \;=\;\frac{\left(1-b^{2}\right)\left(af-p\right)}{2k_{B}\tau_{B}}\;\sigma_{z}, \end{array}$$

where *f*, *g*, and *p* are defined as

(83) $$\begin{array}{c} \begin{array}{cc} f & =\eta_{1}-\eta_{2}-\eta_{3}+\eta_{4},\\ g & =\eta_{1}+\eta_{2}-\eta_{3}-\eta_{4},\\ p & =\eta_{1}-\eta_{2}+\eta_{3}-\eta_{4}, \end{array} \end{array}$$

so that the nonlinear evolution is clearly nontrivial. However, it preserves the zero mean energies of both qubits, and while the overall entropy increases and mutual information partially fades away, it drives the overall state towards a non-dissipative correlated state with maximally mixed marginals. We proved above that signaling is impossible, even though $D_{\rho }^{A}$ depends not only on *a* but also on *b*, and $D_{\rho }^{B}$ on *a*, which agrees with our no-signaling condition in Equation (2).

**Figure 2:**
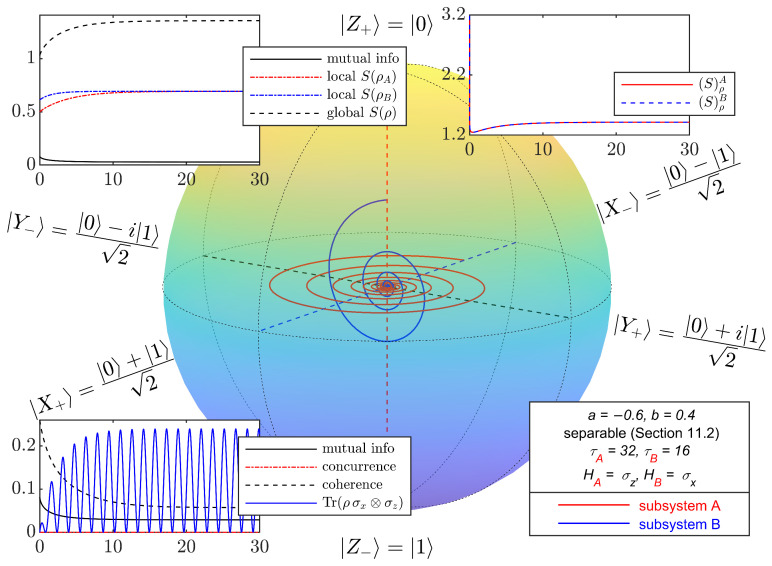
Bloch-ball representation of the time evolutions of the local density operators $\rho_{A}$ and $\rho_{B}$ of non-interacting qubits *A* and *B* with local Hamiltonians $H_{A}=\sigma_{z}$ and $H_{B}=\sigma_{x}$. The evolution follows the steepest locally perceived entropy ascent equation, Equation (39), with ℏ=1 and $k_{B}=1$. The initial state of the composite system AB is the separable, correlated mixed state given by Equation (80) with a=−0.6 and b=0.4. Insets show the time evolutions of global [S(ρ)], local [$S(\rho_{A})$, $S(\rho_{B})$], and locally perceived [${\left(S\right)}_{\rho }^{A}={\left(S\right)}_{\rho }^{B}$] entropies, mutual information [$S\left(\rho_{A}\right)+S\left(\rho_{B}\right)-S\left(\rho \right)$], concurrence—a coherence measure [$\mathrm{Tr}(\rho^{2}H^{2}-\rho H\rho H)$], and the Bell observable $\mathrm{Tr}(\rho \;\sigma_{x}⊗\sigma_{z})$. The local evolutions approach the respective local Gibbs states. Without violating the no-signaling condition, dissipation causes a (non-complete) reduction in mutual information and coherence, while the Bell observable builds up to a steady state oscillation.

Figure 2 shows a Bloch-ball representation of the time evolutions of the local density operators $\rho_{A}$ and $\rho_{B}$ for an initial state in the class considered in this section, Equation (80), with a=−0.6 and b=0.4. The time evolution is computed through a numerical integration of Equation (39). The local evolutions of the two non-interacting qubits approach asymptotically the respective local Gibbs states, i.e., the states of maximum local entropy for the given initial mean local energies, for which Equation (65) reduce to

(84) $$\begin{array}{c} \begin{array}{cc} \Delta (\mathrm{Bln}\left(\rho_{A}\right)) & =-\beta_{A}\;\Delta H_{A},\\ \Delta (\mathrm{Bln}\left(\rho_{B}\right)) & =-\beta_{B}\;\Delta H_{B}. \end{array} \end{array}$$

Mutual information, $S\left(\rho_{A}\right)+S\left(\rho_{B}\right)-S\left(\rho \right)$, and a measure of coherence, $\mathrm{Tr}(\rho^{2}H^{2}-\rho H\rho H)$$=\frac{1}{2}\mathrm{Tr}\left(K^{†}K\right)$, where K=i[H,ρ], are reduced through the SEA dissipation terms but do not vanish. Indeed, the Bell observable, $\mathrm{Tr}(\rho \;\sigma_{x}⊗\sigma_{z})$, which is initially zero, builds up to a periodic oscillation of constant amplitude.

### 11.3. Entangled, Separable, and Correlated Mixed States

**Figure 3:**
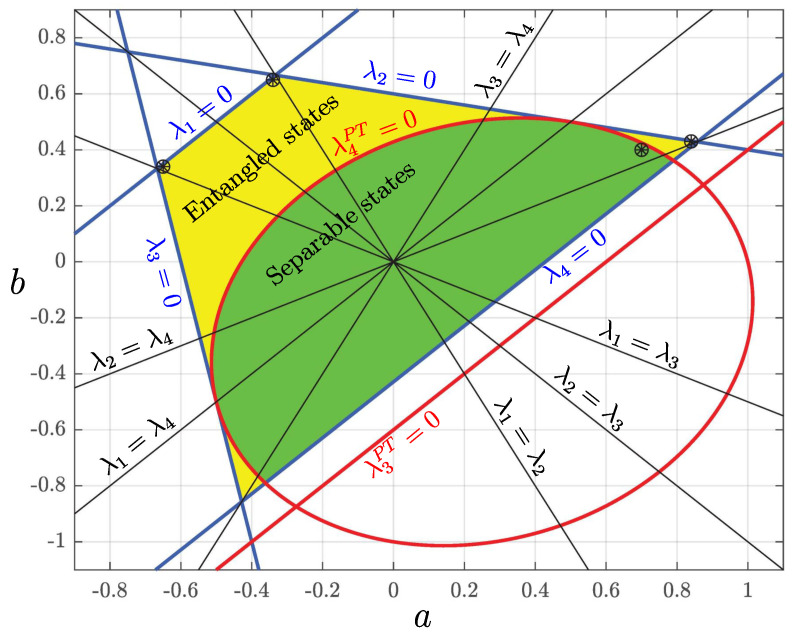
Ranges of admissible values of parameters *a* and *b* for the separable and entangled initial states in the class defined in Section 11.3. The four points denoted with the symbol ⊛ represent the initial states chosen to illustrate the local time evolutions in Figure 4, Figure 5, Figure 6 and Figure 7. The two points near the $\lambda_{1}=0$ line represent the strongly entangled states for which Figure 6 and Figure 7 show a local entropy decrease for one of the subsystems, while the second-law principle of global entropy non-decrease is not violated.

For a slightly more elaborate example that includes entangled mixed states, assume Equation (71) with $a_{x}=a_{z}=a/\sqrt{2}$, $b_{x}=b_{z}=b/\sqrt{2}$, and $c_{x}=c_{y}=c_{z}=2\left(a-b\right)/3$, so that the eigenvalues of ρ and those of its partial transpose are

(85) $$\begin{array}{c} \begin{array}{cc} \lambda_{1}\;=\;\frac{1}{4}\left(1+a-b\right)\;,\; & \lambda_{2}\;=\;\frac{1}{12}\left(3-a-5b\right)\;,\\ \lambda_{3}=\frac{1}{12}\left(3+5a+b\right)\;,\; & \lambda_{4}\;=\;\frac{1}{12}\left(3-7a+7b\right)\;, \end{array} \end{array}$$

and

(86) $$\begin{array}{c} \begin{array}{cc} \lambda_{1}^{PT} & \;=\;\frac{1}{12}\left(3+a-b\right)\;,\;\lambda_{2}^{PT}\;=\;\frac{1}{12}\left(3-5a+5b\right)\;,\\ \lambda_{3}^{PT} & =\frac{1}{12}\left(3+2a-2b+\sqrt{d}\right)\;,\\ \lambda_{4}^{PT} & =\frac{1}{12}\left(3+2a-2b-\sqrt{d}\right)\;, \end{array} \end{array}$$

with $d=25a^{2}-14ab+25b^{2}$. Figure 3 shows the complete set of admissible pairs of values of parameters *a* and *b* for the set of correlated mixed states considered in this example, which encompasses both separable and entangled states. For instance, considering a=−b ($\lambda_{2}=\lambda_{3}$), these states are separable for −3/14≤b≤1/4 and entangled for 1/4<b≤1/2. We compute explicitly each term in Equation (61), also for this class of states, to find

(87) $$\begin{array}{c} \begin{array}{cc} \; & \left\{{\left(S\right)}^{A},\rho_{A}\right\}=-\frac{\left(1-a^{2}\right)\left(f-5bp\right)}{20\sqrt{2}}\left(\sigma_{x}+\sigma_{z}\right)\;,\\ \; & \left\{{\left(S\right)}^{B},\rho_{B}\right\}=\frac{\left(1-b^{2}\right)\left(g+5ap\right)}{20\sqrt{2}}\left(\sigma_{x}+\sigma_{z}\right)\;, \end{array} \end{array}$$

(88) $$\begin{array}{c} \begin{array}{cc} \; & {\left(H,S\right)}_{\rho }^{A}\;=\;-\frac{\left(1-a^{2}\right)\left(f-5bp\right)\omega_{A}}{20\sqrt{2}}\left(h_{x}^{A}+h_{z}^{A}\right)\;,\\ \; & {\left(H,S\right)}_{\rho }^{B}\;=\;\frac{\left(1-b^{2}\right)\left(g+5ap\right)\omega_{B}}{20\sqrt{2}}\left(h_{x}^{B}+h_{z}^{B}\right)\;, \end{array} \end{array}$$

(89) $$\begin{array}{c} \begin{array}{cc} \; & {\left(H,H\right)}_{\rho }^{A}\;=\;\left[1-\frac{1}{2}{\left(h_{x}^{A}+h_{z}^{A}\right)}^{2}a^{2}\right]\omega_{A}^{2}\;,\\ \; & {\left(H,H\right)}_{\rho }^{B}\;=\;\left[1-\frac{1}{2}{\left(h_{x}^{B}+h_{z}^{B}\right)}^{2}b^{2}\right]\omega_{B}^{2}\;, \end{array} \end{array}$$

(90) $$\begin{array}{c} \begin{array}{cc} \; & -\left\{D_{\rho }^{A},\rho_{A}\right\}=-\frac{\left(1-a^{2}\right)\left(f-5bp\right)}{5\sqrt{2}\left[2-{\left(h_{x}^{A}+h_{z}^{A}\right)}^{2}a^{2}\right]k_{B}\tau_{A}}\;\zeta_{A}\;,\\ \; & -\left\{D_{\rho }^{B},\rho_{B}\right\}\;=\;\frac{\left(1-b^{2}\right)\left(g+5ap\right)}{5\sqrt{2}\left[2-{\left(h_{x}^{B}+h_{z}^{B}\right)}^{2}b^{2}\right]k_{B}\tau_{B}}\;\zeta_{B}\;. \end{array} \end{array}$$

Here, *f*, *g*, and *p* are defined as

(91) $$\begin{array}{c} \begin{array}{cc} f & =3\eta_{1}-5\eta_{2}+5\eta_{3}-3\eta_{4}\;,\\ g & =3\eta_{1}+5\eta_{2}-5\eta_{3}-3\eta_{4}\;,\\ p & =\eta_{1}-\eta_{2}-\eta_{3}+\eta_{4}\;, \end{array} \end{array}$$

(92) $$\begin{array}{c} \begin{array}{cc} \zeta_{A} & =\left[1-\frac{1}{2}{\left(h_{x}^{A}+h_{z}^{A}\right)}^{2}a^{2}+\frac{1}{2}\left(h_{x}^{A}+h_{z}^{A}\right)a^{2}\right]\left(\sigma_{x}+\sigma_{z}\right)\\ \; & -\left(h_{x}^{A}+h_{z}^{A}\right)h^{A}\cdot \sigma_{A}\;,\\ \zeta_{B} & =\left[1-\frac{1}{2}{\left(h_{x}^{B}+h_{z}^{B}\right)}^{2}b^{2}+\frac{1}{2}\left(h_{x}^{B}+h_{z}^{B}\right)b^{2}\right]\left(\sigma_{x}+\sigma_{z}\right)\\ \; & -\left(h_{x}^{B}+h_{z}^{B}\right)h^{B}\cdot \sigma_{B}\;. \end{array} \end{array}$$

For example, if the two non-interacting qubits *A* and *B* have local Hamiltonians $H_{A}=\sigma_{z}$ and $H_{B}=\sigma_{x}$, we find

(93) $$\begin{array}{cc} -\left\{D_{\rho }^{A},\rho_{A}\right\} & =-\frac{\left(1-a^{2}\right)\left(f-5bp\right)}{5\sqrt{2}\left(2-a^{2}\right)k_{B}\tau_{A}}\;\sigma_{x}, \end{array}$$

(94) $$\begin{array}{cc} -\left\{D_{\rho }^{B},\rho_{B}\right\} & \;=\;\frac{\left(1-b^{2}\right)\left(g+5ap\right)}{5\sqrt{2}\left(2-b^{2}\right)k_{B}\tau_{B}}\;\sigma_{z}, \end{array}$$

so that again the nonlinear evolution is clearly nontrivial in the sense that the local nonlinear evolution of *A* (*B*) does not depend only on $\rho_{A}$ ($\rho_{B}$), despite being no-signaling.

**Figure 4:**
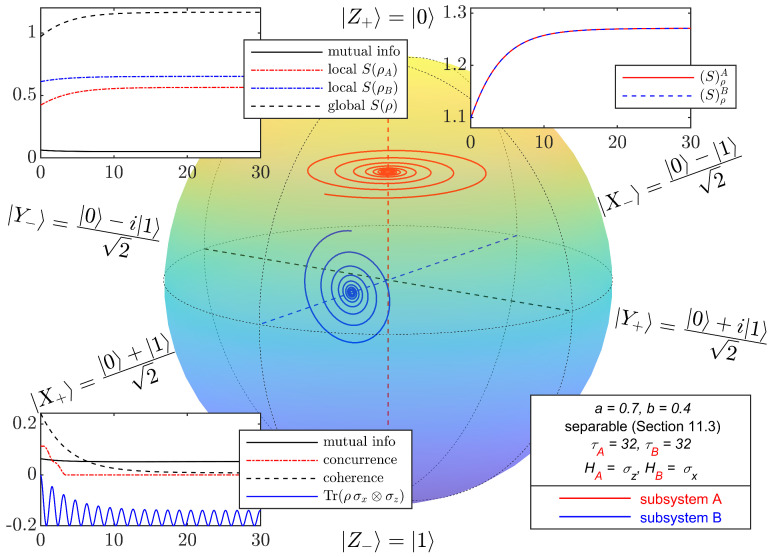
Bloch-ball representation of the time evolutions of the local density operators $\rho_{A}$ and $\rho_{B}$ of non-interacting qubits *A* and *B* with local Hamiltonians $H_{A}=\sigma_{z}$ and $H_{B}=\sigma_{x}$, resulting from numerical integration of the steepest locally perceived entropy ascent equation of motion, Equation (39). The initial state of the composite system AB is the separable, correlated mixed state in the class of states defined in Section 11.3 with a=0.7 and b=0.4. The local evolutions approach the respective local Gibbs states. Without violating the no-signaling condition, dissipation causes a (non-complete) reduction in mutual information and coherence, while the Bell observable converges to a steady state oscillation. Insets are similar to Figure 2.

**Figure 5:**
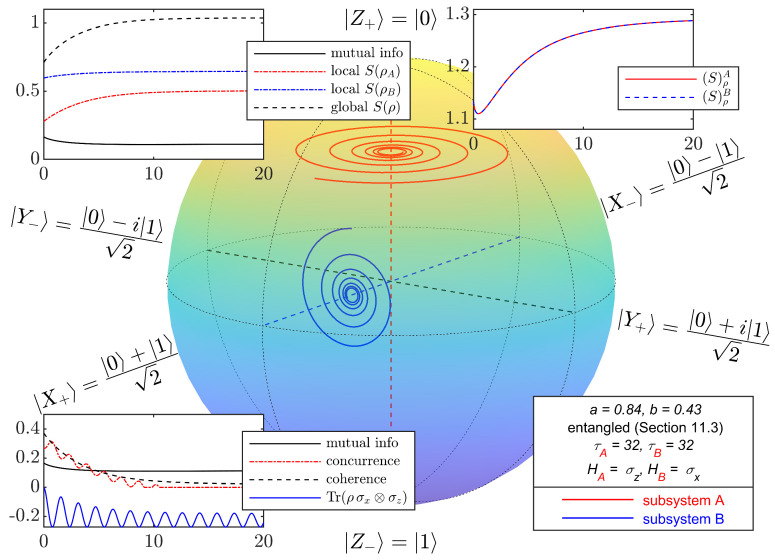
Bloch-ball representations of the time evolution of two non-interacting qubits, obtained from numerical integration of Equation (39) with initial entangled mixed states from the class defined in Section 11.3. The values of (a,b) are (0.84,0.43). Due to entanglement, local evolutions approach limit cycles with local entropy lower than the corresponding local Gibbs states. Insets are similar to Figure 2.

**Figure 6:**
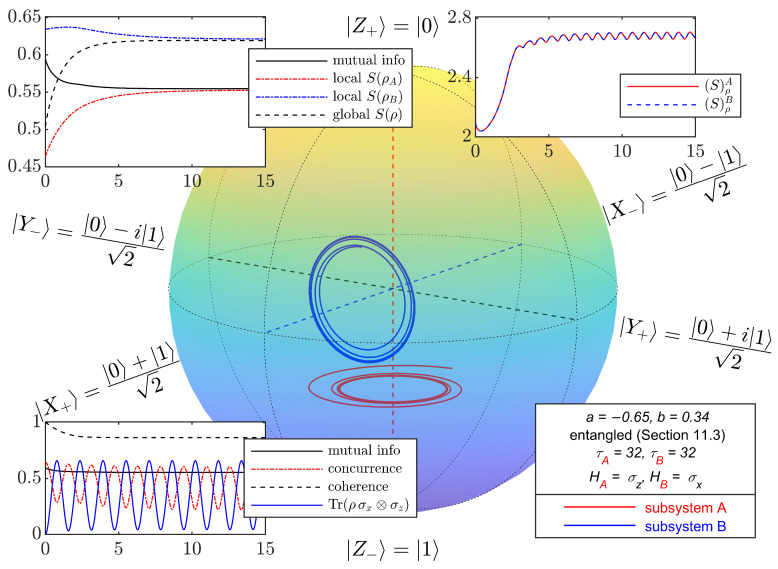
Bloch-ball representations of the time evolution of two non-interacting qubits, obtained from numerical integration of Equation (39) with initial entangled mixed states from the class defined in Section 11.3. The values of (a,b) are (−0.65,0.34). Due to entanglement, local evolutions approach limit cycles with local entropy significantly lower than the corresponding local Gibbs states. Strong entanglement leads to a local entropy decrease in subsystem *B* and *A*, respectively, without violating the global entropy non-decrease dictated by the second law. Insets are presented in Figure 2.

**Figure 7:**
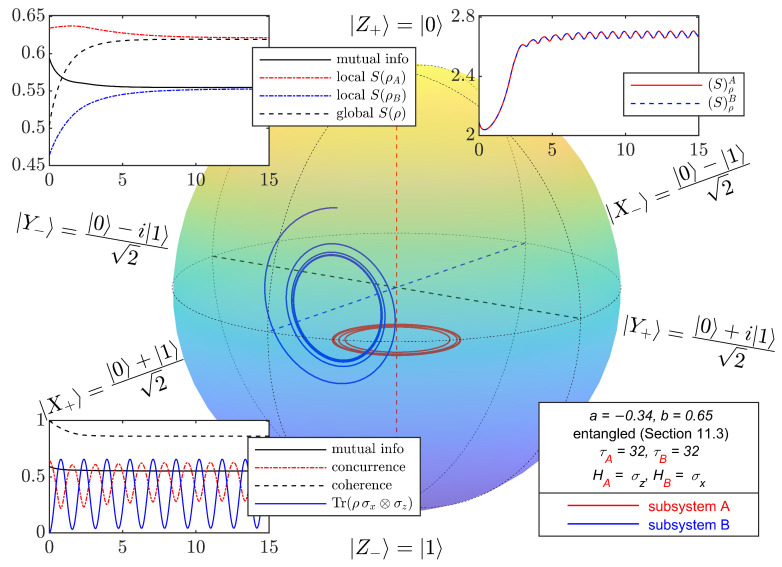
Bloch-ball representations of the time evolution of two non-interacting qubits, obtained from numerical integration of Equation (39) with initial entangled mixed states from the class defined in Section 11.3. The values of (a,b) are (−0.34,0.65). Due to entanglement, local evolutions approach limit cycles with local entropy significantly lower than the corresponding local Gibbs states. Strong entanglement leads to a local entropy decrease in subsystem *B* and *A*, respectively, without violating the global entropy non-decrease dictated by the second law. Insets are similar to Figure 2.

For initial states in the class considered in this section, with parameter values *a* and *b* corresponding to the four points in Figure 3, Figure 4, Figure 5, Figure 6 and Figure 7 depict the typical time evolution of the local density operators $\rho_{A}$ and $\rho_{B}$ in the local Bloch-balls. These results are obtained from the numerical integration of the steepest locally perceived entropy ascent equation of motion, Equation (39), for non-interacting subsystems *A* and *B*.

Whereas, for a separable initial state, Figure 4 shows that the local states approach the respective local Gibbs states, it is clear from Figure 5, Figure 6 and Figure 7 that the presence of entanglement does not allow the local states to approach the respective local Gibbs states. Indeed, the local evolutions approach non-dissipative unitary limit cycles where $\{D^{A},\rho_{A}\}\;=\;0$ and $\{D^{B},\rho_{B}\}\;=\;0$, and therefore, Equation (65) is satisfied. Without violating the no-signaling condition, dissipation causes a (non-complete) reduction in mutual information and coherence, while the Bell observable $\mathrm{Tr}(\rho \;\sigma_{x}⊗\sigma_{z})$ and concurrence converge to steady state oscillations.

## 12. Conclusions

In this work, we have explored the no-signaling principle and its implications for nonlinear dynamics in quantum theory. We examined the measure-theoretic representation of mixed ensembles by providing a consistent framework for handling nonlinearities and describe ensemble mixing unambiguously. This approach also offers a conceptual basis for developing nonlinear quantum dynamical models for non-equilibrium systems and addressing unresolved questions about individual quantum states. Additionally, we reviewed the original philosophical motivations underlying the steepest entropy ascent (SEA) nonlinear evolution law, which trades linearity for strong thermodynamic consistency.

Our analysis demonstrates that the SEA formalism provides a valid framework for describing evolution or designing master equations. These can be applied in nonlinear extensions of quantum mechanics (QM) or in phenomenological models of open quantum systems within quantum thermodynamics. Our key focus was the no-signaling principle. We introduced a criterion for no-signaling based on local perception operators (LPOs), a generalization of the traditional density-operator-based definition. Unlike the conventional assumption that local evolution depends solely on local properties, SEA formalism incorporates the local effects of preexisting correlations and coherence through LPOs. Our detailed examination of the foundations and properties of LPOs highlights their potential as key tools for constructing signaling-free, nonlinear, and non-local non-equilibrium dynamical models, even outside of the SEA framework.

By inherently respecting the no-signaling condition, the SEA formalism upholds the principle of causality and aligns with the axiomatic paradigm proposed by Popescu and Rohrlich [86] for quantum theories. Furthermore, the SEA approach addresses conceptual challenges such as the Schrödinger–Park paradox, offering insights into a thermodynamically consistent integration of quantum mechanics and non-equilibrium thermodynamics. Related to the no-signaling property, our discussion of LPOs and supporting numerical examples strengthens the conjecture—supported by prior numerical results from Cano-Andrade et al. [25]—that SEA evolution is universally compatible with decoherence principles. Specifically, SEA evolution ensures that correlations and entanglement cannot build up between non-interacting systems but can decay partially or completely, a process mediated via LPOs.

An intriguing implication of SEA formalism is its compatibility with thermodynamic principles while allowing for scenarios where, under strong entanglement, the local entropy of a subsystem may temporarily decrease during the evolution, without violating the no-signaling condition or the global entropy non-decrease dictated by the second law. We emphasize that, differently from the Lindbladian ‘bottom-up’ approach in which quantum thermodynamic second law(s) emerge from statistical approximations of the linear unitary dynamics induced by system-environment interactions, the SEA ‘top-down’ approach builds strong second-law compatibility directly into the nonequilibrium dynamics variational principle, constrained by the phenomenological details about the system’s structure, subsystems’ interactions, and environmental effects. However, because of the inherent nonlinearity of the SEA formalism, the solution becomes more complex, and a full analytical calculation can become intractable [27]. Additionally, the relaxation times τJJ¯ remain heuristic parameters, which are not derived from first principles and need to be tuned with respect to a given experimental set-up.

We hope this study provides a foundational basis for exploring applications of SEA modeling in quantum computing. Our comprehensive approach and observations aim to stimulate constructive discussions and inspire further studies, particularly in developing thermodynamically consistent models for non-equilibrium processes. These findings have potential applications in modeling the dynamics of quantum systems, advancing quantum technologies, and contributing to the framework of quantum thermodynamic resource theories, even beyond the SEA paradigm.

## Data Availability

The data presented in this study are available on request from the corresponding author. There are MATLAB codes that are used to generate plots. If reasonable request is made; we will provide them.
